# Bone Grafts in Dental Medicine: An Overview of Autografts, Allografts and Synthetic Materials

**DOI:** 10.3390/ma16114117

**Published:** 2023-05-31

**Authors:** Maria Pia Ferraz

**Affiliations:** 1 Departamento de Engenharia Metalúrgica e de Materiais, Faculdade de Engenharia da Universidade do Porto, 4200-465 Porto, Portugal; mpferraz@fe.up.pt or mpferraz@ineb.up.pt; 2 i3S—Instituto de Investigação e Inovação em Saúde, Universidade do Porto, 4099-002 Porto, Portugal; 3 INEB—Instituto de Engenharia Biomédica, Universidade do Porto, 4099-002 Porto, Portugal

**Keywords:** bone defects, bone reconstruction, bone graft, synthetic bone substitutes, dental bone substitutes

## Abstract

This review provides an overview of various materials used in dentistry and oral and maxillofacial surgeries to replace or repair bone defects. The choice of material depends on factors such as tissue viability, size, shape, and defect volume. While small bone defects can regenerate naturally, extensive defects or loss or pathological fractures require surgical intervention and the use of substitute bones. Autologous bone, taken from the patient’s own body, is the gold standard for bone grafting but has drawbacks such as uncertain prognosis, surgery at the donor site, and limited availability. Other alternatives for medium and small-sized defects include allografts (from human donors), xenografts (from animals), and synthetic materials with osteoconductive properties. Allografts are carefully selected and processed human bone materials, while xenografts are derived from animals and possess similar chemical composition to human bone. Synthetic materials such as ceramics and bioactive glasses are used for small defects but may lack osteoinductivity and moldability. Calcium-phosphate-based ceramics, particularly hydroxyapatite, are extensively studied and commonly used due to their compositional similarity to natural bone. Additional components, such as growth factors, autogenous bone, and therapeutic elements, can be incorporated into synthetic or xenogeneic scaffolds to enhance their osteogenic properties. This review aims to provide a comprehensive analysis of grafting materials in dentistry, discussing their properties, advantages, and disadvantages. It also highlights the challenges of analyzing in vivo and clinical studies to select the most suitable option for specific situations.

## 1. Introduction

In addition to autologous bone, several other materials are used in dentistry and oral and maxillofacial surgeries to replace or repair bone defects. The selection of the best material depends on several factors, including tissue viability, size, shape, and defect volume [1,2].

Bone grafting is a common procedure in dental medicine used in various situations. Some common clinical dental medical procedures in which bone grafts are needed are dental implants, ridge augmentation, sinus lift, socket preservation, and periodontal surgery [3,4,5,6,7,8]. Dental implants are artificial tooth roots that are placed into the jawbone to support dental prosthetics, such as crowns, bridges, or dentures. Sufficient jawbone volume and density are crucial for successful implant placement. If a patient lacks adequate bone in the implant site, a bone graft may be necessary to augment the area and provide a solid foundation for the implant [4]. When a tooth is extracted, the surrounding bone may shrink or resorb over time. Ridge augmentation is a procedure in which bone grafts are used to rebuild and restore the height and width of the jawbone ridge. This procedure may be performed to create a suitable foundation for dental implants or to improve the appearance of the gumline [8]. The maxillary sinuses are air-filled spaces located above the upper jawbone. If the upper jawbone has insufficient height or volume to support dental implants in the posterior region, a sinus lift procedure may be required. In this procedure, the sinus membrane is lifted, and bone graft material is placed between the sinus membrane and the jawbone to increase the bone height [6]. When a tooth is extracted, socket preservation techniques can be used to minimize bone loss in the empty socket and preserve the bone volume for future dental implant placement. A bone graft is typically placed in the socket after the tooth extraction to fill the void and maintain the bone structure [7]. In some cases of advanced periodontal disease, bone loss can occur around the teeth, leading to tooth mobility and eventual tooth loss. Bone grafts may be used in periodontal surgery to regenerate and restore the lost bone around the affected teeth, promoting stability and preventing further tooth loss [3].

Under healthy conditions, small bone defects manage to regenerate spontaneously; however, extensive bone defects or loss, pathological fractures, and bone infection due to periodontal problems or systemic diseases can influence bone healing and regeneration, requiring surgical intervention and the choice of a substitute bone [1,9,10].

Extensive bone defects are usually treated with autologous bone taken from the iliac crest or the calvaria. Autologous bone contains osteogenic cells capable of synthesizing new bone and its structure serves as a scaffold, making this procedure the gold standard of bone grafting. However, this procedure has some disadvantages [1,2,11].

To avoid complications, other bone substitutes are often used in medium and small-size defects and include allografts (human bone other than the patient’s own (e.g., extracted from cadavers)), xenografts (bone from animals other than human species), and synthetic materials with osteoconductive properties that can be reabsorbed by the body, releasing substances that contribute to the formation of new bone (e.g., ceramics, bioactive glasses, polymers, synthetic hydroxyapatite (HA)) [12,13].

Bone grafts should have specific requirements in order to be used and have optimal performance: (i) unlimited supply without compromising the donor area, (ii) promote osteogenesis, (iii) no host immune response, (iv) rapid revascularization, (v) stimulate osteoinduction, (vi) promote osteoconduction, and (vii) be completely replaced with bone in quantity and quality similar to that of the host [2,14,15].

Osteoinduction is defined as the process by which osteogenesis (i.e., new bone formation from osteocompetent cells in connective tissue or cartilage) is induced. Osteoconduction is defined as the process of bony ingrowth from local osseous tissue onto surfaces. Osteogenic materials are defined as those which contain living cells and are capable of differentiation into bone [16].

The purpose of this review is to provide a comprehensive overview of the grafting materials that can be used in dentistry, discussing their properties, advantages, and disadvantages, enlightening the problems of analysing in vivo and clinical studies in order to choose the best option in a particular situation.

## 2. Bone Grafts in Dental Medicine

There are several materials available for bone grafts and the selection of the ideal material depends on a number of factors, such as material availability, defect size, size, shape and volume of the graft, biomechanics, handling, cost, ethical issues, biological characteristics, and associated complications [2]. Among the available options in the area of bone regeneration, the gold standard remains autogenous bone, due to its osteoinductive and osteogenic capabilities. All other materials (allograft, xenograft, and synthetic biomaterials) have limitations, which must be taken into account, depending on their use [2]. These categories will be briefly described below.

### 2.1. Autografts

Autografts are the gold standard materials for bone grafts in the field of medicine and dentistry due to the fact that these materials have many of the requirements considered optimal for a bone graft as they are biocompatible, non-toxic, osteogenic, osteoinductive, and osteoconductive [17,18].

These advantages are fundamental for fast and efficient bone regeneration, mainly in defects considered of critical size (>5 mm), since the vascularization is reduced in the centre of these defects [17,18]. Healing time is also dependent on the material used, with autologous bone being the most rapidly vascularized and, therefore, the most osteogenic of all materials currently available [19,20]. It is important to emphasize that the combination of cortical and medullary bone is one of the most advantageous in the area of bone regeneration, since it unites two important characteristics: the support and mechanical resistance of the cortical bone and the osteogenic function of the medullary bone [20].

However, this procedure has some disadvantages, namely, the uncertain prognosis and surgery at the bone removal site as well as the sequelae that may occur in the process, such as the risk of infections. Additionally, the quantity and quality of the donor’s bone may be insufficient, due to age-related problems or disorders that may affect the patient’s medical condition (e.g., metabolic diseases, osteoporosis, and diabetes) [1,2,11].

In dentistry, this type of procedure is only used in critical cases, such as jaw reconstruction, congenital bone defects, tumours, and bone defects larger than 5 mm, due to the limited amount of intraoral bone and the need for an extra procedure to remove bone from another area, requiring hospitalization, a hospital environment, and a multidisciplinary team [21]. With the need for an extra surgery to remove autogenous bone, the risks inherent to any surgery increase: pain, infection, scars, in addition to extra costs with hospitalization and a multidisciplinary team [21,22,23]. Autologous bone, although still considered the best option, has been replaced over the years by other materials, with the aim of reducing patient morbidity, treatment costs, and surgical time, as well as the postoperative period.

### 2.2. Allografts

Allografts are derived from individuals within the same species. After extensive screening, these grafts are carefully selected, processed, and preserved in bone banks. Allografts can originate from living donors or cadaveric bone material after being processed to eliminate immune responses and prevent transmitting infectious diseases. These grafts are available in different shapes and sizes, including cortical, cancellous, or cortico-cancellous grafts [24].

Allografts, despite being used with some frequency in regenerative treatments in some areas of medicine, these materials are not one of the first-choice materials in dentistry. There is still some controversy regarding their osteoinductivity, as well as their risk of immune rejection, blood incompatibility, and disease transmission [2,12,18].

Allogeneic materials are considered a source of type I collagen and morphogenetic proteins (BMPs), which give them osteoinductive capabilities. However, although they originate from the human species, they have different genetic compositions, which raises controversy about immunological rejection, blood compatibility, and transmission of diseases or tumour cells [2,12,18]. Considered osteoinductive and osteoconductive, they do not have osteogenic properties, and their processing ends up reducing their biological and mechanical characteristics [2,12,18].

Although with some advantages similar to autogenous bone and greater availability, allogeneic materials have a high processing cost, in addition to the already mentioned disadvantages regarding disease transmission, immunological rejection, and religious issues [2,12,18].

### 2.3. Xenografts

Materials of animal origin, xenografts, are widely used in dentistry, being well-documented materials studied for more than three decades [24]. Their osteoconductivity comes from their inorganic structure, composed mainly of HA, obtained through the removal of all organic components [25]. Xenografts can be of the most diverse origins, the most used being those of bovine and porcine origin; however, other origins include horses, coral exoskeleton, and eggshells, among others [26,27,28,29]. One of the advantages of xenogeneic materials is the similarity of their chemical composition to human bone, with a calcium/phosphate ratio of 1.67, identical to that of human bone [30]. Their disadvantage comes from ethical, religious, and health issues, such as the risk of disease transmission [2,31].

Xenografts are the materials most used by dentists. Their effectiveness is very well documented in several comparative studies with other materials, mainly with autologous bone [2,20,32,33].

One of the xenogeneic materials on which there are more publications and which is also well known by dentists is Bio-Oss^®^. Bio-Oss^®^ is obtained from bovine HA; one of its main characteristics is its similarity in chemical composition with human HA. Its calcium/phosphate ratio of 1.67 is identical to that found in human bone [18].

Materials from other sources, such as equine, porcine, coral exoskeletons, and even eggshells, have been studied and commercialized [26,27,28,29,34,35,36,37,38].

Each material has specific characteristics, but in general, among the advantages of these materials, it is possible to mention their low cost, great availability, and osteoconduction [2].

Consisting entirely of inorganic bone, with no organic or cellular content, some materials, such as Bio-Oss^®^, are also considered osteoinductive, information that conflicts with some authors, who consider that osteoinduction occurs when there is cellular material, such as morphogenic proteins, growth factors, or some living material in the composition of the bone graft [2,18].

Due to this osteoinductive characteristic, materials of animal origin have been the subject of controversies and discussions about their use in humans. As a natural material, it is possible that they retain some of their original characteristics after processing, such as some cellular activity that gives them the osteoinductive characteristic [31,39].

Although companies that market bones of xenogeneic origin guarantee that their products are completely free of any organic material, some plastic surgeons have detected proteins, such as collagen, in Bio-Oss^®^ after orthognathic surgery [40]. In another study, reaction to foreign bodies, which consisted of multinucleated cells encapsulated within inorganic bovine bone particles, was reported after histological analysis [25]. These findings contribute to the controversy about the transmission of diseases that can occur when these materials are used. As sporadic as these cases may be, it is important to inform the patient about this risk and alternatives.

If there is no organic component in xenogeneic materials, their osteoinductive capabilities are questionable and, although many studies confirm their osseointegration characteristics, other materials have been the subject of studies, in order to overcome the ethical and religious issues of xenogeneic materials, as well as to improve the manipulation capabilities, to facilitate the procedure for doctors and dentists [20,28,30,39,41,42,43,44,45,46]. In addition to the issues mentioned above, these materials require experienced handling. As they are particulate materials, they require the use of a membrane to keep the static particles at the defect site and prevent the connective tissue from invading the area that must be remodelled, which makes the procedure more complicated [22,29].

### 2.4. Synthetic Materials

The role of synthetic bone materials is to promote bone regeneration. Synthetic materials have several advantages concerning the surgical method necessary for obtaining autogenous material: biocompatibility, osteoconduction, injectability, moldability, easy manipulation, minimally invasive procedure, scar reduction (since only the affected area is surgically treated and only one surgery is required), in addition to the decreased risk of infection and other complications [47,48,49]. Another advantage is their wide availability, since the material can be easily manufactured in scale, unlike autogenous, allogeneic, or xenogeneic materials.

The growing demand for materials for bone reconstruction has stimulated research in the area of biomaterials, in order to supply the scarce source of autogenous and allogeneic bone available [14]. Several bioceramic materials have been developed as an alternative, and several studies—both experimental and clinical—have demonstrated the osteoconductive properties (materials that facilitate infiltration through the bone surrounding the defect) of these materials when used for medium and small bone defects, increasing the bone crest for implant placement, bone defects due to periodontal disease, and maxillary sinus elevation [43,50,51,52,53]. Within the group of ceramics, materials based on calcium phosphate are extensively studied and frequently used as bone grafts due to their compositional similarity with natural bone, with their HA demonstrating excellent biocompatibility. In addition to the granular form, these materials can be manipulated in the form of a paste, which reduces application time and, mainly, improves moldability to the defect [47,50,54,55]. The use of calcium phosphates for larger defects is restricted due to their lack of osteoinductivity; therefore, there are several studies in order to meet this need [56].

It is important to emphasize that synthetic biomaterials do not have osteoinductive properties (the potential to induce bone formation), considered ideal for the formation of new bone. For this reason, the use of these materials still brings some disadvantages when used in bone defects of critical size, which encourages constant research and the inclusion of other components in an attempt to improve their performance. Other materials can be incorporated into scaffolds of synthetic or xenogeneic origin with the aim of improving their osteogenic properties. Growth factors, cellular content, autogenous bone, and therapeutic elements are some of the materials studied and incorporated into these materials with the aim of increasing biological performance and improving the quantity and quality of new bone [2,57,58,59,60]. This area of study, called tissue bioengineering, is based on key elements, which form the triad: (i) scaffold or carrier material; (ii) biological components (growth factors, drugs); (iii) cells [61].

In Table 1, bone graft categories as well as their source of origin or chemical constitution with examples of commercially available products are exemplified.

## 3. Types of Synthetic Materials

To decide which material is most appropriate for a given procedure, it is necessary not only to have a good understanding of the biological function (osteogenesis, osteoinduction, and osteoconduction) of each material but also to consider the patient condition, as this is an essential criterion for the incorporation of any bone graft. Bone grafts are evolving and undergoing innumerable changes and there has long been talk of synthetic bone grafts and bone substitutes to the detriment of autologous, allogeneic, or even xenogeneic grafts [18,22,23].

Bioactive cements are considered good alternative bone substitutes, due to their moldability, self-hardening, and osteoconductivity. However, although these biomaterials are already widely used, they still need to improve their mechanical properties [55]. Regarding synthetic bone grafts, scaffolds give mechanical support and serve as a substrate where osteoblastic or osteoprogenitor cells can adhere, proliferate, and differentiate for the formation of new bone. They can also be used as carriers for other materials, with the addition of growth factors or drugs, or mixed with other types of bone grafts to increase or improve bone formation [2,81,82,83]. The most studied biomaterials among synthetic bone grafts are cements based on calcium phosphate, calcium phosphate ceramics, calcium sulphate, bioactive glasses, and polymers [18,20,22,28,48,50,58,84,85].

### 3.1. Calcium Phosphate Cements

Calcium-phosphate-based materials have been used since the 1980s in the fields of dentistry and orthopaedics and are currently commercially available in a wide variety of compositions [86]. Calcium phosphate cements (CPCs) have several advantages, including being bioactive, allowing for large-scale manufacturing, easy handling, and injectability to adapt to irregularly shaped bone defects, in addition to not having the inherent risks of autogenous and allogeneic grafts, such as donor site morbidity and risk of infection. Furthermore, their biocompatibility and proximity to bone composition make CPCs good candidates for use in bone regeneration [45,47].

Among CPCs, there are two main groups: those of brushite that have a shorter hardening time and those of apatite that have a longer hardening time. Apatite is formed from tetracalcium phosphate (TTCP) or α-TCP, while brushite is a by-product of β-TCP or monocalcium phosphate monohydrate (MCPM). The difference between these two by-products derives from the fact that cements that form brushite absorb more water in their mixing and hardening reaction, while apatite absorbs little or no water. Brushite-based CPCs react and harden much faster than apatite CPCs; therefore, to satisfy the necessary clinical requirements of cement application during surgery, the setting time of CPCs materials based on brushite must be increased, while the setting time of apatite-based CPCs should be reduced [47]. Through absorbing more water, the cements that turn into brushite have less resistance to tension, compression, and shear [87,88].

### 3.2. Calcium Phosphate Ceramics

Ceramic materials based on calcium phosphate (CP) can be found in the form of granules or blocks with none or different porosities [89,90,91] and include HA, tricalcium phosphate (α-TCP and β-TCP), biphasic calcium phosphate (BCP), and amorphous calcium phosphate (ACP), among others [18,22,92].

#### 3.2.1. Hydroxyapatite

Hydroxyapatite’s (Ca_10_(PO_4_)_6_(OH)_2_) (HA’s) composition has a great similarity with the mineral part of the bone and, for this reason, it has been widely documented for its ability to promote bone growth through its osteoconductive mechanism without causing local or systemic toxicity, inflammation, or undesirable immune reactions [91,92,93]. All these advantages make this material very useful in the area of bone repair in dentistry, such as in the treatment of periodontal defects, alveolar crest augmentation, and maxillary sinus elevation [27,93,94,95].

HA nanoparticles, with particle size smaller than 100 nm in at least one direction, have greater surface activity and an ultrafine structure, very similar to the mineral found in hard tissues, which stimulates their use in the area of bone regeneration. In addition to chemical similarities with the mineral phase of bone, they also have excellent biological properties [96,97,98].

Another advantage of this material, shown in several studies, would be its affinity with certain osteogenic and anti-resorptive molecules, which can be used to create reservoirs for growth factors, antibiotics, or medication to inhibit osteoclasts [2,99].

#### 3.2.2. Tricalcium Phosphate

Beta-tricalcium phosphate (β-TCP) is sintered at a temperature lower than ~1125 °C and has the advantage of thermodynamic stability in a biological environment and being more resorbable than HA at room temperature.

Alpha-tricalcium phosphate has been gaining great attention in the area of biomaterials as a raw material, due to its properties such as injectability and biodegradation. This material remains stable when, after the sintering process, it is cooled to room temperature [100]. Despite having similar chemical composition, α and β TCP have considerable differences in their structure, density, and solubility, which determine their biological characteristics and specific clinical applications. Since α-TCP is more soluble and reactive than β-TCP, its ultrafine powder is the mostly used in the preparation of cements for bone repair, to improve the moldability and injectability of the cement [100].

#### 3.2.3. Calcium Sulphate

Calcium sulphate hemihydrate (CaSO_4_·1/2H_2_O), also known as plaster of Paris, has been used since the mid-1920s as a bone filler. The dissolution properties of this material have been used in the study and development of carrier materials for molecules that improve bone quantity or quality or as a carrier for drugs such as antibiotics [92].

#### 3.2.4. Bioactive Glasses

Bioactive glasses (BGs) are a group of synthetic materials based on silica, calcium, and disodium oxide. As calcium and silicate ions are progressively released from the material, they interact with surrounding cells and thus have properties that allow it to bind to bone [17]. They have unique properties when compared to other ceramics such as HA and TCP, namely, the formation of an amorphous layer on their surface where proteins, collagen, fibrin, and growth factors connect. This surface contributes to the bone reconstruction process, as it is chemically and structurally equivalent to the bone mineralization phase [17]. Depending on their chemical composition, BGs differ in their bioactivity and resorption. In vivo, this material showed good osteoconductivity and appears to promote new bone growth on its surface, demonstrating a balance between intramedullary bone formation and material resorption [28,101]. Some studies demonstrate little or no inflammatory reaction, foreign body reaction, or fibrous encapsulation of the material when bioactive glasses are used [17,34]. Due to their osteoconductive properties, composition, and in vitro and in vivo results, BGs have been a group of constant study for use as a bone substitute [17,34].

#### 3.2.5. Polymers

Studies involving polymers are based on the search for materials that can support and maintain space for the period necessary for the formation of new bone and, after this period, can be degraded and eliminated by the host organism [102]. The most studied materials currently are polymers based on glycolic acid and lactic acid, also known as PLGA and PLA, respectively. These polymers can be easily degraded by the organism, but the lack of mechanical resistance, as well as their low osteoconductivity, make this material unsuitable to be used alone as a scaffold [103]. Its degradability is a great advantage and, therefore, this material has been incorporated into CPC- or BG-based materials, with the aim of improving the handling of these materials as well as injectability [47,102]. These polymers have also been used to improve the osteogenic properties of other materials, in addition to being extensively studied as carriers of molecules, such as growth factors or drugs [104].

In Table 2 examples of trademarks, composition, and mechanisms of action described by manufacturers of synthetic materials used as bone grafts.

## 4. Clinical Applications

In dentistry, the use of synthetic materials for bone repair has gained more and more space, especially in the field of surgery and periodontics. Bone cements have gained notable attention due to their injectability and moldability qualities, where there are several comparative studies that suggest that their use brings advantages in relation to other synthetic materials [27,41,122].

In dentistry, the use of synthetic materials has gained ground in several surgeries, namely for maxillary sinus lift, periodontal defects, and bone crest augmentation. Most of these procedures aim to improve the quantity and quality of bone for the insertion of dental implants [94].

Rehabilitation with implants can be a problem if there is insufficient bone or if that bone is of poor quality. When there is a bone defect, it is difficult to achieve the primary stability necessary for implant placement and osseointegration [123]. It can also be difficult to achieve this primary stability in the region close to the maxillary sinuses, where the amount of cancellous bone is greater than cortical bone [21,39].

The use of synthetic material as a bone graft for maxillary sinus elevation has been well documented and studies point out that some materials have demonstrated good degradation and bone integration in humans after three months after implantation, with no evidence of significant differences when implant placement occurs after three or six months of bone augmentation [123].

As in any surgery, the choice of material must be based on the characteristics of the defect; its shape, size, and location; as well as the type of intervention and the characteristics of the material. There is still no consensus on the time required for material integration and osseointegration, as this time depends on the type of material, the amount that is not completely degraded, and the way the implant will be placed, depending on the torque force and the primary implant stability [35].

## 5. Difficulties in Analysis of In Vivo and Clinical Trials Results

There are many materials for bone repair available on the market and the choice of the dentist should be based on multiple factors, which include tissue viability as well as the size, shape, and volume of the defect [2].

Many studies have been published on the most varied types of materials, but there is still no consensus among dentists on the best option. What is agreed in this area is the use of autologous bone whenever possible, given the proven and superior osteoinductivity and osteogenesis of this material. Many studies prove its better efficacy in the regeneration of bone tissues when compared to other materials [21,34,124].

In dentistry, the use of autologous bone is restricted to very specific cases, due to its complicated procedure, invasive technique, and need for a hospital environment and multidisciplinary team, in addition to cost [2]. Among the materials most used in dentistry are materials of xenogeneic origin and synthetic materials. There are many articles published about the advantages of each group, as well as their proven biocompatibility and osteoconductivity [27,34].

Publications demonstrate great variability in the way of developing their studies. The chosen animal model, sex, type of defect, surgical conditions, healing time, chosen material, and the way the material is manipulated or applied are some of the variables that make comparative analysis difficult. Although in vivo experiments follow criteria and protocols, there is no single methodology in what concerns to sample selection, sample allocation, measurement of analysed variables, or reading of results.

For the study of materials for bone regeneration, an understanding of each animal specimen and specific characteristics of the bone is necessary, such as bone microstructure and composition, the properties of bone formation and remodelling, and other characteristics that should be similar to human bone as much as possible [125]. This comparison is exemplified in Table 3 [125].

Although mice are widely used animal models, they are significantly different from human bone on many levels. As a result, regeneration resulting from the implantation of biomaterials can hardly be used as an assumption of similar behaviour in humans [125].

Rabbits are also animals which are widely used in medical research. As with rodents, a great disadvantage is related to their size, which does not allow the implantation of many materials in the same model [125]. The bone structure of the rabbit also differs from human bone; however, as an advantage, it is possible to mention bone maturation within six months after birth, rapid skeletal change, as well as rapid bone turnover. These conditions allow results in a shorter period of time in in vivo tests [125]. In terms of bone composition, the animal models that most closely resemble humans, anatomically and physiologically, are dogs and pigs. Pigs bear great resemblance to human bone, but as the pig increases in size over time, this makes it difficult to control and handle the animal [125].

Clinical studies in humans are at the top of the medical research pyramid, as they are the most complex and expensive, but provide the most reliable answer. In order to reach testing in humans, the material must have been tested over several years in other animal models, which means that newly discovered technologies cannot yet be tested in this group. Nine publications with studies carried out in humans [26,27,30,34,39,41,122,126,127] are going to be analysed.

In order to exemplify the difficulty of comparing the different alternatives, some examples of studies will be reported according to the animal model. Given that allografts are not alternatives widely used in dentistry, comparison between xenografts and synthetic materials was chosen.

### 5.1. Studies in Rodents

In the case of the experiments in rats, Poehling [128] detected greater bone regeneration when synthetic material was used with the incorporation of growth factors in the repair of the bone defect. This study encompassed 60 specimens studied in the group of synthetic materials and 72 specimens in the group of xenogeneic materials [128].

The study [128] evaluated a material called MD05, which is a combination of beta-tricalcium phosphate coated with a growth factor called rhGDF-5. MD05 is being studied as a bone graft material for dental and facial applications. The goal of the study was to compare the bone-healing properties of MD05 with commercially available bone substitutes. The researchers created skull defects in rats and implanted different materials in the defects, including MD05, bovine bone mineral, and other bone substitutes. After 6 weeks, they analysed the samples and found that MD05 showed significantly better bone regeneration compared to the other materials. The defects filled with MD05 had more new bone formation, less fibrous tissue, and better overall bone repair. MD05 also showed the ability to bridge the defect completely and support normal bone marrow growth. The growth factor rhGDF-5 in MD05 seemed to play a role in promoting bone growth.

The xenogeneic materials (Bio-Oss^®^, Wolhusen, Switzerland) and PepGen^®^, Boston, MA, USA) showed limited bone growth compared to the two synthetic materials analysed in the study (with and without rhGDF-5) with growth restricted to the margins of the bone defect.

Based on these results, the researchers concluded that MD05 is a promising bone substitute for dental and facial applications, as it demonstrated superior bone regeneration compared to conventional materials.

### 5.2. Studies in Minipigs/Guineapigs

Four studies using minipigs and/or guineapigs were chosen as examples to be described [18,129,130,131]. Three different materials were analysed in the synthetic group HA (Ostim^®^, Ankara, Turkey), β-TCP (Ceros^®^, New York, NY, USA), and macroporous biphasic calcium phosphate MBCP. The xenogeneic group had only one material used, Bio-Oss^®^ [18,129,130,131].

The study by Jensen [129] used β-TCP (Ceros^®^) and Bio-Oss^®^. β-TCP presented less residual material when compared to Bio-Oss^®^ after eight weeks of analysis. However, the use of β-TCP induced the formation of a connective tissue layer with the presence of macrophage cells. Moreover, at the junction between β-TCP and newly formed bone, signs of β-TCP dissolution were observed, indicating dissolution and not direct resorption. Higher bone formation was also observed with β-TCP when compared to the xenogeneic material after 8 weeks.

Concerning Bio-Oss^®^, it was almost completely incorporated into the bone, increasing bone density raising better biological support. No reduction or reabsorption of the xenogeneic material was observed, but, as mentioned, its incorporation into the new bone was observed, therefore suggesting that the osteoclastic cells found at the site performed an important role in cleaning the surface of the particles for future osseointegration of the xenogeneic material. Unlike Bio-Oss^®^, Ceros^®^ demonstrated resorption in all stages analysed and greater bone formation. Osteoclastic cells were not found along the material but in nearby areas, with the function of phagocytizing dissolved particles.

Regarding Busenlechner’s [130] study, the authors claimed that the limited number of samples available for simulations limits the direct comparison of different bone substitutes. The main target of this study was to develop a preclinical model for guided bone regeneration that enables testing of various bone substitutes in a specific type of bone defect. The authors reported that up to eight titanium hemispheres can be placed on the minipigs’ calvaria and filled with materials to be tested. Bio-Oss (a deproteinized bovine bone mineral), Ostim (an aqueous paste of synthetic nanoparticular hydroxyapatite), and Osteoinductal (an oily calcium hydroxide suspension) were tested using this model for 6 and 12 weeks. The results showed that hemispheres filled with Bio-Oss and Ostim exhibited nearly complete bone formation, consistent with their documented osteoconductive properties. However, Osteoinductal did not demonstrate osteoconductive properties; instead, it led to progressive resorption of the host bone.

In another study by the same author [131], three different areas of the defect were analysed. HA based material (Ostim^®^) and Bio-Oss^®^ were analysed, and this study [131] tested a different quantitative method to analyse bone volume formation based on a bi-dimensional analysis, while other studies analyse bone formation in three dimensions through computed micro-tomography (μCT). In both materials, the highest concentration of formed bone was found close to the bone wall of the defect (three-walled defect). Ostim^®^ demonstrated large bone formation close to the defect wall, with less material visualized when compared to Bio-Oss^®^ in the same region. This area of intense bone formation close to the bone wall of the defect with intense vascularization also demonstrates the osteoconductivity of the synthetic material, given the different degree of bone formation between both materials. Ostim^®^ promoted greater bone formation close to the defect and in an area up to 3 mm from the defect. Bio-Oss^®^ promoted less formation close to the defect wall and within 3 mm of the defect area, but it was possible to observe bone formation beyond 5 mm from the defect, which confirms its osteoconduction.

In another study on guinea pigs, by Yazdi [18], a material based on HA and β-TCP (MBCP) was used, with a polymeric carrier phase which creates a better-handling gel, and compared with Bio-Oss^®^. It was observed in that study that with MBCP, bone formation was distributed throughout the defect and not just at the margins. Bone formed throughout the defect in the path of the material. It was also possible to observe material reabsorption signs, with the presence of osteoids. No significant inflammatory reaction was observed for both MBCP and Bio-Oss^®^. In this study, excellent angiogenesis and osteogenesis were observed in the MBCP, both on the surface of the defect and in depth. This material demonstrated better osteoconductivity compared to Bio-Oss^®^. This could have been caused, as mentioned in the study, by the stability of the MBCP gel, compared to the loose particles of Bio-Oss^®^. Even with stabilized Bio-Oss^®^, the removal of particles from the site was inevitable. In this study, a membrane was not used to cover Bio-Oss^®^. As observed in the study, this instability of the material at the defect site can inhibit bone formation and lead to the formation of fibrous tissue during healing.

Studies with minipigs continue to be used in recent years [132,133]; however, translation of the information into humans is still difficult [134]. Notwithstanding, there is recent information attesting that minipigs and humans have similar bone formation and remodeling rates [134].

Several animal studies have been published reporting on dental bone graft efficacy; however, animal models fail to mimic the complexity of the oral environment and uniqueness of the alveolar bone [135]. In a recent review [135], the minipig intraoral dental implant model was evaluated and a meta-analysis was conducted to estimate osseointegration and crestal bone remodelling. They conclude that the minipig intraoral dental implant model effectively demonstrates osseointegration and alveolar bone remodelling, similar to what is observed in humans. However, the quality of reporting in the studies included in this review was generally low, suggesting the need for improved reporting standards in future research.

### 5.3. Studies in Rabbits

Concerning rabbits, five studies are going to be described in detail [36,44,45,136,137].

In the study by de Souza [136], the comparison between β-TCP (Cerasorb^®^) and Bio-Oss^®^ showed similar bone formation between these materials after 60 days of experiment, as well as residual material in both cases. The percentage of soft tissue found was significantly higher in the β-TCP group after 60 days. Although angiogenic expression was similar for both materials, Bio-Oss^®^ allowed greater osteoblastic differentiation than the synthetic material.

Kruse [137] used synthetic HA with silica oxide (Nanobone^®^) and demonstrated similar bone repair when compared to a xenogeneic material (Bio-Oss^®^) in rabbit calvaria. Statistical differences were not significant in this study. This indicates the similar ability of these materials to regenerate bone. To summarize, in that study [137], results suggest that synthetic hydroxyapatite/silica oxide granules yield comparable outcomes to a standard xenogeneic bovine mineral in terms of bone formation and bridging non-critical size defects.

The study by Schmidlin [45] used β-TCP and BCP in the group of synthetic materials and compared them with Bio-Oss^®^. This study was performed without placing periosteum or membrane to cover the materials. In this study, the superiority of the xenogeneic material in bone formation in rabbits was verified. In this study, BCP was more efficient in centripetal bone formation when compared with TCP. After 16 weeks, all of the β-TCP material was resorbed and the greatest degradation of the material was found at the periphery of the defect in the particles involved in new bone. BCP showed no degradability after 16 weeks. ABBM showed the greatest material degradation in the regenerated tissue.

Lee [36] conducted a study using an eggshell-based (EHA) material, which demonstrated superiority when compared to HA. An organized lamellar island was found where EHA was used, in addition to a lower percentage of residual material, with a statistically significant difference in relation to HA. This study showed a lower mean percentage of bone formed with the use of HA.

Lambert [44] used rabbits that underwent bilateral sinus elevation using three different types of grafts: the xenograft (BHA), beta-tricalcium phosphate (β-TCP), or biphasic calcium phosphate (BCP). In this study, researcher demonstrated that after 6 months of observation, synthetic materials (β-TCP and BCP) had a higher percentage of bone formation compared to the xenograft (BHA). No multinucleated cells were found in the tissue generated via xenograft, and only lamellar bone was in close contact with the material particles. β-TCP was no longer visible after the six months of study, which suggested its almost complete resorption. It was possible to notice that the reabsorption of this material is dependent on the observation time [45,136]. While β-TCP resorption occurred almost entirely, BCP still remained with visible particles. In the case of BCP, osteoblastic activity and osteoid tissue was found along the trabecular bone, suggesting active (osteogenic) remodelling. Multinucleated cells were also found next to BCP, as well as capillaries, which were not located either in xenogeneic material or β-TCP. The percentage of soft tissue found in the xenogeneic specimens was significantly lower than the percentages found in the other materials. This study concluded that osteogenesis was observed with all three biomaterials in this specific animal model. After 6 months, there were significant differences in biomaterial resorption rates among the three groups. The β-TCP showed the highest resorption rate. After 6 months, bone closely interacted with the BHA particles, forming a composite network, whereas BCP particles were frequently surrounded by soft tissue. The study concluded that future investigations in humans should consider longer follow-up periods.

Recent studies using rabbits as models for bone graft evaluation [138,139] continue to have problems concerning translating the information into humans.

### 5.4. Studies in Dogs

Four studies in dogs were chosen to be described as examples [22,32,42,140]. In the four studies described, seven different materials were analysed in the synthetic group (β-TCP, β-TCP + mesenchymal cells (MSC), HA (Apaceram), BCP/MSC, BCP and HA with particles of different sizes). The xenogeneic group analysed four different materials (Bio-Oss^®^, Bio-Oss^®^ + MSC, ABBM without commercial brand, with particles of different sizes) [22,32,42,140].

In the study by Carvalho [32], materials with granules of different sizes were compared. The best result concerning new bone formation was observed with xenografts of small particles (150–200 μm). Xenografts with large particles (300–329 μm) also obtained better results compared to the synthetic material with large particles of 300 μm. In this study, xenogeneic materials had a rougher surface than HA, which may have positively affected the adhesion of osteoblasts and their proliferation. Since the materials in this study were experimental, the authors suggest that other factors such as the higher crystalline phase of the materials and little amorphous phase may have affected the results.

Jafarian [140] demonstrated the highest percentage of new bone formation with mesenchymal cells incorporated into BCP (Kasios^®^, L’Union, France) when compared to Kasios^®^ without cells and Bio-Oss^®^ with or without cells. After six weeks, little osteoclastic activity and resorption was observed for both Bio-Oss^®^ + MSC and Kasios^®^ + MSC. Although without significant difference, the percentage of new bone formed by Kasios + MSC was higher than that registered with Bio-Oss^®^ + MSC. A similar result was observed when comparing Kasios^®^ and Bio-Oss^®^ materials without the presence of MSC, where a higher percentage of new bone prevailed in the synthetic material.

Kim [42] studied a material based on synthetic HA in comparison to Bio-Oss^®^. The percentage of bone formation with HA was significantly higher than that of Bio-Oss^®^ and the percentage of soft tissue found in the HA group was lower than in Bio-Oss^®^, with 16 weeks of observation. It was also verified in this study that both materials were poorly reabsorbed by the body during this period. However, HA maintained a more appropriate geometric structure for vessel formation and bone growth than Bio-Oss^®^. Particulate xenogeneic material seems to lose most of its porosity and interconnectivity in the process of transforming the bone block into particles, while in the synthetic material this porosity and structure of the material can be planned and modulated, which can be beneficial for bone remodelling.

A study in dogs by Vahabi [22] investigated the incorporation of mesenchymal cells into BCP and compared this material with Bio-Oss^®^ and with the synthetic material alone. This study also demonstrated the role of mesenchymal cells in the formation of trabecular bone, with more soft tissue involved. Although there was no statistically significant difference between the materials, Bio-Oss^®^ and BCP were shown to form less trabecular and cortical bone than BCP incorporated with cellular material. The same result was found in a study by Jafarian [140], with large bone formation in the synthetic material with incorporation of mesenchymal cells. The highest percentage of residual material was found in Bio-Oss^®^, and the highest reaction to foreign bodies was found with this material, but without significant differences for the other materials.

Several attempts have been made to find animal models to test dental bone grafts; however, the mandible model in beagle dogs is currently the best animal model for vertical bone augmentation [141,142]. However, the use of the canine model is limited by socio-ethical factors [142].

### 5.5. Studies in Humans

Scarano studied 96 patients who underwent maxillary sinus lift. After an observation period of 6 months, the study concluded that all analysed materials are biocompatible and did not show inflammatory signs [34]. The materials that obtained the greatest amount of bone formation were the xenogeneic derivatives (Bio-Oss^®^ and PepGen^®^) in comparison with bioactive glass and a polymer-based material (Fisiograft^®^ sponge). Fisiograft^®^ is a polymer and, as such, has a faster degradation than particulate materials. Considerable variation in the quality and quantity of formed bone has been reported in the literature in relation to the type of material used and biopsy time [122,126,128,143].

Cordaro [126] also analysed elevation of the maxillary sinus after an observation period of 180–240 days and concluded that BCP demonstrated greater formation of new bone when compared to xenografts. Briefly, lateral sinus augmentation was used, with grafting using BCP and xenogens. The difference between the materials is due to the fact that xenografts have the amount of bone formed covering its particles, with a greater amount of residual material compared to BCP. With regard to bone formation, both have a similar amount of new bone, with similar histological appearance, which indicates that both are good materials for maxillary sinus elevation and implant placement. This study is multicentre, with different clinicians, but with a standard protocol, which included two groups with similar characteristics, treated with an identical surgical protocol. This is an important consideration, as there are several factors that can interfere with the results of the study. The study concluded that there are no statistically significant differences in the amount of new bone formed between the materials analysed when used for maxillary sinus lift, but there are statistically significant differences in the residual material left by the xenograft, which is surrounded by new bone and in relation to the soft tissue formed, which was statistically higher in the case of BCP.

Simunek [41] analysed bilateral maxillary sinus elevation after nine months of observation. The particle size of both materials ranged between 1000–2000 μm. The study concluded that xenografts obtained the highest percentage of bone formation compared to β-TCP, but it was not possible to conclude the amount of residual material.

Froum [127] also compared bone regeneration in bilateral maxillary sinus lift, using only two materials, and observed greater formation of new bone with BCP. Briefly, following elevation of the lateral sinus walls, one material was placed in the right sinus and the other material was placed in the left sinus. From the histological point of view, the two materials proved to be osteoconductive after an observation period that varied between six and eight months. The BCP material demonstrated a slight increase in the amount of bone formed. The best results regarding bone formation were obtained after eight months of observation. New bone was observed adjacent to and around the material particles, while in xenogeneic material, the particles were surrounded by a greater or lesser amount of new bone and osteoid.

Crespi [26] also compared materials but used tooth sockets in his study. In 15 patients, 45 extractions were performed and 30 sockets were filled with magnesium-enriched hydroxyapatite (MHA) or porcine bone (PB). After the four-month observation period, the histomorphometric analysis of the biopsies revealed absence of inflammatory cells in both materials. A small difference in percent bone volume was found with PB versus MHA, but no significant differences. The authors concluded that the clinical performance of both materials was similar.

Iezzi [27] also analysed materials in relation to bilateral maxillary sinus lift. The author compared four different materials randomly applied to 15 patients (30 maxillary sinuses). All analysed xenogeneic materials, with the exception of calcium carbonate, had a higher percentage of new bone formation compared to BCP. The microporosity of the analysed materials allowed the growth of new bone and blood vessels inside the pores of the partially resorbed particles, mainly in Algipore^®^. The study concluded that all analysed materials have similar characteristics and can be used for the maxillary sinus lift procedure.

Pettinicchio [122] also compared several materials in relation to the elevation of the maxillary sinus and concluded that Engipore^®^ and Bio-Oss^®^ produced a similar percentage of new bone and that none of the materials tested was completely absorbed after six months of observation. Bio-Oss^®^ showed that its particles appear osteointegrated in the formed trabecular bone. This information is in line with other studies [44,126,127,129]. The synthetic material Engipore^®^ showed a tendency to concentrate the bone apposition within the microporosities. The mineralized tissue appeared to be formed mainly of collagen fibres, randomly oriented with some areas of osteoid tissue. The lowest percentage of residual material was also found in Engipore^®^, which agrees with other authors [44,45,126,129]. Petinicchio’s study also concluded that the amount of bone formed depends on the type of biomaterial and the number of residual particles was inversely proportional to the amount of bone formed. The material derived from porcine bone (PepGen^®^) demonstrated a lower percentage of formed bone and a greater amount of residual material.

In the study by Lindgren [39], patients underwent bilateral maxillary sinus lift, which made it possible to compare both materials used within the same biological conditions, contrary to the study by Kurkco [30]. The BCP and xenograft materials were used and analysed after three years of follow-up, but despite the initial number of participants (11 patients), at the end of this observation period it was possible to use only five biopsies of each material, given the difficulty in obtaining tissue with the necessary characteristics around the implants that were placed. The histomorphometric study demonstrated greater new bone formation in biopsies containing ABBM, but without significant differences between the materials. Greater inflammatory reaction was seen in BCP biopsies, which may affect the bone remodelling process, causing osteolytic or osteosclerotic lesions. However, inflammatory cells also have the ability to increase the differentiation and activity of osteoblasts and osteoclasts, but in most cases this result has more impact on bone loss than on its remodelling [144]. The study concluded that, despite the greater bone formation of the xenograft, the choice of biomaterial does not influence the survival of the implants.

Another recent study, by Kurkco [30], analysed the xenograft (Boneplus-xs^®^ and the synthetic material β-TCP (Kasios^®^). Both materials, with particle sizes between 1000–2000 μm, were used for maxillary sinus lift. A negative point of this study comes from the fact that it was not possible to compare both materials in the same patient. Patients underwent unilateral maxillary sinus lift and, therefore, the biological conditions of each patient are considered in the study result. The mean observation period in this study was 6.5 months, when the biopsy was performed. Both materials were mixed with the patient’s own blood and neither material received a membrane. Bone formation was statistically significantly different, with greater bone formation found in patients receiving xenogeneic material. A greater amount of residual material was found with β-TCP, but without significant differences in relation to xenogeneic material. Kurkco’s article confirms other studies, which suggest that the configuration of the xenogeneic particles results in better osteoconductivity, since the chemical composition, crystal and particle size, material porosity, and surface texture of the particles have been reported as influencers of the performance [32,41].

Only Froum [127], Crespi [26], and Lindgren [39] studied materials from different sources in the same patient. Although the conditions of the spaces filled by the material in the same patient may differ in size, studies that use identical methods allow a more reliable evaluation and comparison of the healing response of the analysed materials. Among the three studies, only the study by Froum resulted in a higher percentage of bone volume formed when using a synthetic material, BCP. This same study confirmed other similar results [39,41,126] when it pointed to a direct relationship between the maturation time of the synthetic material and bone formation, being more visible bone remodelling after eight months of study, while xenogafts did not demonstrate this trend, with no changes in bone formation at six and eight months of observation.

## 6. Conclusions

The growing demand for materials for bone reconstruction has stimulated research in the area of biomaterials, in order to supply the scarce source of autogenous and allogeneic bone available, as well as to extinguish the issue related to the transmission of diseases that is generated in the use of bone from xenogeneic origin.

Synthetic materials have gained ground among medicine and dentistry professionals due to their ease of handling, injectability, self-hardening, and because they are a reproducible material. Their large-scale, planned, and modulated manufacture is another advantage of these materials, since studies agree that bone formation is directly linked to the composition of the material, size, shape, and porosity of the particles, which is difficult to be controlled in the production of xenogeneic materials.

New synthetic bone substitutes have demonstrated good biological behavior in bone formation compared to bone of xenogeneic origin. The initial phases of every study include animals of reduced size, such as mice and minipigs. As research progresses, larger animals may be included, with human studies being the final phase of the investigation. Therefore, new materials are still in this phase of research.

Experiments on patients are the final phase of studying a product and materials with recent technology are not yet used or commercialized or are under analysis. Thus, it is understandable that, in clinical trials, studies have a statistically significant result in favor of materials of xenogeneic origin, which have been on the market for over 30 years and are very well documented, with their effectiveness proven.

Nanotechnology is creating opportunities for the development of more bioactive bone substitutes, which release substances that improve cellular biological performance, activate reparative cascades, or inhibit osteolytic processes. The best results in new bone formation within the analysed studies were found when incorporating mesenchymal cells and growth factors.

In the future, comparative studies in humans may reveal whether this evolution of synthetic bone substitutes will be beneficial for better regeneration and remodeling with greater bone quantity and quality than materials of xenogeneic origin, especially in critical defects larger than 5 mm in diameter and bone defects that require a material with greater mechanical resistance.

## Figures and Tables

**Table 1:** Bone graft categories their source of origin or chemical constitution with examples of commercially available products.

Graft Category	Origin/Chemical Constitution	Advantages	Disadvantages	Examples of Commercially Available Products	References
Autografts		Osteogenic Osteoinductive Osteoconductive No disease transmission No immunogenicity	Donor site morbidity Limited quantity Needs for general anaesthesia and hospitalization		
		
		
Allografts		Osteoinductive Osteoconductive Moderate availability	Risk of disease Transmission Immunogenicity	DBX^®^	[62]
				DynaBlast^®^	[63]
				DynaGraft^®^	[64]
				Grafton™	[65]
				Opteform^®^	[23]
				OsteoSponge^®^	[62]
				Puros^®^	[66]
				Raptos^®^	[24]
Xenografts	Bovine	Osteoconductive High availability	Risk of disease Transmission Immunogenicity	Algipore^®^	[24]
	Porcine			Bio-Oss^®^	[67]
	Equine			Endobon^®^	[23]
	Coraline			Cerabone^®^	[68]
	Algae			Gen-Os^®^	[69]
				OsteoBiol^®^	[70]
				Pro Osteon^®^	[71]
				THE Graft™	[24]
				Biocoral^®^	[72]
Synthetic bone substitutes	Calcium phosphate	Osteoconductive Availability		BonePlast^®^	[73]
	Hydroxyapatite			Cortoss^®^	[74]
	Calcium carbonate			Eurobone^®^	[75]
				EasyGraft™ crystal	[76]
				EasyGraft™ classic	[77]
	Calcium sulphate			Vitoss^®^	[24]
	Bioactive glasses			Guidor^®^	[78]
	Polymers			HydroSet^®^	[79]
				IngeniOs^®^	[24]
				B-OstIN^®^	[24]
				PerioGlass^®^	[24]
				Straumann^®^	[24]
				BioGran^®^	[80]

**Table 2:** Trademarks, composition, and mechanisms of action described by manufacturers.

Trademarks	Composition	Mechanisms of Action Described by Manufacturers	Reference
BonePlast^®^	Calcium Sulphate with/without HA granules	Osteoconductive; Resorbable	[73]
Conduit™	100% β-TCP	Osteoconductive; Resorbable	[105]
OpteMx™	HA/TCP biphasic	Osteoconductive; Resorbable; Osteogenic and osteoinductive when mixed with medullary bone	[2]
Integra Mozaik™	80% β-TCP, 20% collagen type I	Osteoconductive; Resorbable	[106]
MasterGraft™	Biphasic Calcium Phosphate (15% HA, 85% β-TCP)	Osteoconductive; Resorbable	[107]
NovaBone^®^	Bioactive silicate	Osteoconductive; Resorbable	[108]
Vitoss^®^	100% β-TCP/80% β-TCP + 20% collagen/70% β-TCP, 20% collagen, 10% bioactive glass	Osteoconductive; Resorbable; Osteogenic and osteoconductive when mixed with medullary bone	[24]
Calceon^®^ 6	Calcium sulphate	Osteoconductive; Resorbable	[105]
Norian^®^ SRS^®^	Calcium Phosphate	Osteoconductive; Resorbable	[109]
MIIG X3	Calcium sulphate	Osteoconductive; Resorbable	[110]
Osteoset^®^	Calcium sulphate	Osteoconductive; Resorbable	[111]
Pro Dense™	75% calcium sulphate, 25% calcium phosphate	Osteoconductive; Resorbable	[112]
Pro-STIM™	50% calcium sulphate, 10% calcium phosphate, 40% demineralized bovine bone	Osteoconductive; Resorbable; osteoinductive	[113]
CopiOS^®^ Bone	Biphasic calcium phosphate and collagen type 1	Osteoconductive; Resorbable; Osteogenic and limited osteoinductive when mixed with medullary bone	[114]
Cerasorb^®^	100% β-TCP	Resorbable	[115]
Straumann Bone Ceramic^®^	Biphasic calcium phosphate (60% HA/40 β-TCP)	Osteoconductive; Able toinduce vascularization and osteoblast migration	[24]
EasyGraft™ crystal	Biphasic calcium phosphate (60% HA/40 β-TCP)	Resorbable; Osteoregenerative	[76]
EasyGraft™ classic	Pure β-TCP phase (>99%)	Resorbable; Osteoregenerative	[77]
ENGIpore^®^	Synthetic HA	Osteoconductive	[116]
Apaceram^®^	Synthetic HA	Osteoconductive	[117]
Ostim^®^	Pure HA phase	Osteoconductive; Resorbable	[118]
Ceros^®^ TCP	100% β-TCP	Osteoconductive; Resorbable	[119]
Calciresorb^®^	96% β-TCP, 4% HA	Osteoconductive; Resorbable	[120]
Fisiograft^®^	HA and polyethylene glycol (PEG)	Partially resorbable	[121]

**Table 3:** Comparison between human and animal bone: four attributes (+ low similarity; ++ moderately similar, +++ very similar).

	Rodents	Pigs	Rabbits	Dogs
Macrostructure	+	++	+	++
Microstructure	+	++	+	++
Bone composition	+	+++	++	+++
Bone remodeling	+	+++	+	++
